# The complete mitochondrial genome of the South American freshwater crayfish, *Parastacus nicoleti* (Crustacea, Decapoda, Parastacidae)

**DOI:** 10.1080/23802359.2019.1699457

**Published:** 2019-12-12

**Authors:** Su-Jung Ji, Dong-Ha Ahn, Gi-Sik Min

**Affiliations:** a Department of Biological Sciences, Inha University, Incheon, South Korea;; b SOKN Institute of Ecology and Conservation, Yangpyeong-gun, Gyeonggi-do, South Korea

**Keywords:** Chilean freshwater crayfish, mitogenome, *Parastacus nicoleti*, phylogenetic analysis

## Abstract

We determined the complete mitochondrial genome (mitogenome) sequence of a Chilean freshwater crayfish, *Parastacus nicoleti* (Philippi, 1882). This is the first complete mitogenome sequence of a non-Australian crayfish belonging to the family Parastacidae. The complete mitogenome of *P. nicoleti* is 20,894 bp in length and contains 13 protein-coding genes (PCGs), 22 transfer RNAs (tRNAs), two ribosomal RNAs (rRNAs) and a putative control region (CR). In the phylogenetic analysis, freshwater crayfishes were clearly divided into two monophyly groups, Northern and Southern Hemisphere groups. The *P. nicoleti* exhibited a sister-group relationship with all other Australian parastacid crayfishes.

Freshwater crayfishes, which belong to the infraorder Astacidea Latreille, 1802 are geographically divided into two groups, Northern and Southern Hemisphere crayfishes (Ahn et al. 2006). The Northern Hemisphere crayfishes belong to two families, Astacidae and Cambaridae, while the Southern Hemisphere crayfishes belong to one family, Parastacidae.

The genus *Parastacus* Huxley, 1879, belonging to the family Parastacidae, consists of 13 South American crayfish species (Crandall and De Grave 2017; Ribeiro et al. 2017; Huber et al. 2018). *Parastacus nicoleti* (Philippi, 1882) is a crayfish endemic to Chile. To date, 32 complete mitochondrial genomes (mitogenomes) of parastacid crayfishes, which were all obtained from Australian crayfishes, have been deposited in the GenBank. Therefore, the complete mitogenome of *P. nicoleti*, which was determined in the present study, is the first complete mitogenome sequence of a non-Australian crayfish belonging to the family Parastacidae.

*Parastacus nicoleti* was collected from a swamp at Reumén, Valdivia, Chile (39°59′S, 72°49′W). The total genomic DNA was extracted from the muscles of an ethanol preserved crayfish claw using the DNeasy Blood & Tissue Kit (Qiagen, Hilden, Germany). PCR amplification, nucleotide sequencing, and gene annotation for the complete mitogenome sequence were performed according to Kim et al. (2012). The extracted total DNA was deposited in the DNA collection of the National Institute of Biological Resources, Incheon, South Korea (Deposit no. NIBRGR0000609055). For phylogenetic analysis, we used the sequences of 13 protein-coding genes (PCGs). A maximum-likelihood tree was constructed using IQ-TREE v1.6.8 based on the TIM2 + F + R4 model and 1,000 ultrafast bootstrap replicates (Nguyen et al. 2015; Hoang et al. 2018; Kalyaanamoorthy et al. 2017).

The complete mitogenome of *P. nicoleti* (GenBank accession number: MN617015) is 20,894 bp in length and contains 13 protein-coding genes, 22 transfer RNAs (tRNAs), two ribosomal RNAs (rRNAs) and a putative control region (CR). Mitogenome of *P. nicoleti* is the largest in the family Parastacidae to date, with six long AT-rich intergenic sequence regions and a long CR (1,359 bp).

For the phylogenetic analysis, 22 mitogenome sequences which represented four extant astacidean superfamilies (Astacoidea, Parastacoidea, Nephropoidea, and Enoplometopoidea) were used. *Homarus americanus* (Nephropoidea) and *Enoplometopus debelius* (Enoplometopoidea) were used as an outgroup. In the maximum-likelihood tree (Figure 1), freshwater crayfishes were clearly divided into two monophyly groups, Northern and Southern Hemisphere groups. *Parastacus nicoleti*, the only parastacid species from South America, exhibited a sister-group relationship with all other Australian parastacid crayfishes. More mitogenome sequences from South America and Madagascar would facilitate the clear definition of relationships among the Southern Hemisphere crayfishes.

**Figure 1.**
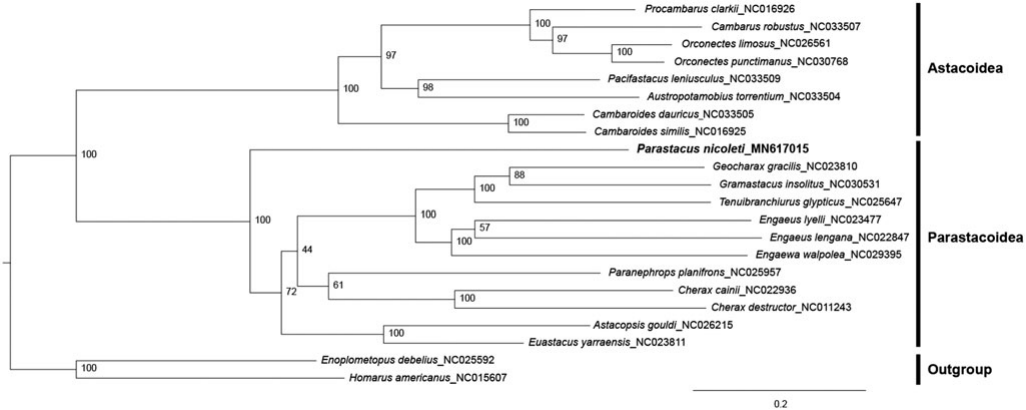
A maximum-likelihood tree within the infraorder Astacidea. The bootstrap supports are shown on each node.
